# Delayed Presentation of Trichobezoar with Small Bowel Obstruction

**Published:** 2011-03-10

**Authors:** Naima Zamir, Jamshed Akhtar, Soofia Ahmed

**Affiliations:** Department of Pediatric Surgery, National Institute of Child Health Karachi, Pakistan

**Keywords:** Trichobezoar, Intestinal obstruction, Child

## Abstract

Small bowel obstruction is a common surgical emergency but trichobezoar as an etiology, rarely reported. A seven year old school going female child presented with acute intestinal obstruction with a palpable and mobile mass in the abdomen. At exploration, a 10 cm long trichobezoar was found in the distal ileum which was removed through enterotomy. Postoperative course remained uneventful. Further probing revealed that child used to eat her own scalp hairs at the age of 2 years and the habit persisted for about 18 months which resulted in alopecia at that time. Later on she started showing normal behavior.

## INTRODUCTION

Trichobezoar is a ball of swallowed hairs usually found in stomach. It is not an uncommon condition. It usually is limited to stomach or may have its extension into small bowel in a form of tail - the Rapunzel syndrome. Presence of trichobezoar in small bowel without a trace in stomach is a rare occurrence [1, 2]. Delayed presentation after many years of presence of trichobezoar in alimentary tract is a rare event. We are reporting a case of delayed presentation of trichobezoar which presented with acute small bowel obstruction.

## CASE REPORT

A seven-year-old female child presented with acute onset of colicky abdominal pain and non bilious vomiting for the preceding three days. There was no history of fever and passage of round worms in stool. On examination the child was alert and of normal built. Abdomen was not distended. It was soft with a palpable elongated mobile minimally tender mass. A provisional diagnosis of mesenteric / ovarian cyst was made.

X-ray abdomen was suggestive of mechanical obstruction. Ultrasound showed mass to be non cystic. Abdominal lymph nodes were not enlarged and other viscera were reported as normal.

At laparotomy an intraluminal mass was found in distal ileum. It was compressible. Rest of bowel and stomach were normal. An enterotomy was made and a large trichobezoar was removed. It was 10 cm long (Fig. 1). The enterotomy was then closed.

**Figure:**
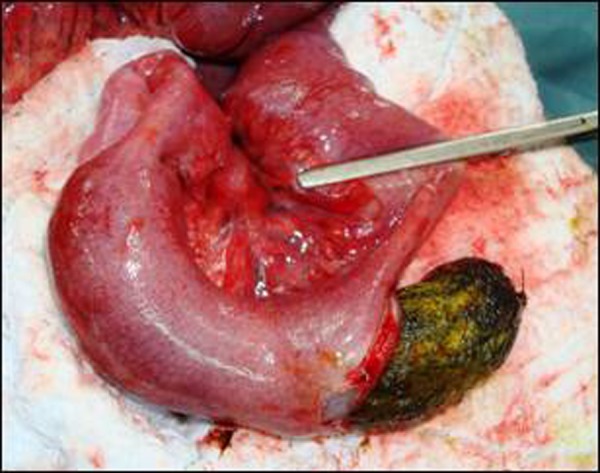
Figure 1: Enterotomy and removal of Trichobezoar

The postoperative course remained uneventful. Further probing revealed that child had habit of eating her own hairs from the age of 2 years which persisted for about 18 months. Alopecia was reported at that age. Presently she is a normal looking average built school child interested in surroundings and quite social; but parents were concerned about her jealous behavior as to her younger sister. Child was also consulted with a psychiatrist who following a session declared that at present she has no ailment and being jealous of younger sibling is a normal phenomenon.

## DISCUSSION

 Trichobezoar is a unique rare condition predominantly of childhood and adolescence. It is a black, glistening, foul smelling mass, made up of hairs present in the alimentary tract, commonly in stomach. There is usually a preceding history trichotillomania (pulling own hairs) followed by trichophagia (ingestion of hairs).

Not all the cases of trichotillomania have trichophagia nor all the trichophagia develop trichobezoar. Although the exact cause of trichotillomania is not clear certain psychosocial, behavioral, and biological theories have been proposed like childhood trauma, stress and neurochemical imbalances (like of serotonin) [3, 4].

Hairs are non absorbable or digestible and also due to smooth slippery texture not easily pass out of the alimentary tract. They remain stuck in the folds of alimentary tract and usually jumbled up in stomach. This bunch of hair can have extension in to distal bowel as a result of peristaltic propulsion. It may get detached as satellite in distal intestine with main part in the stomach. There are recurrent episodes of non specific pain in abdomen, loss of appetite, vomiting and weight loss. Alopecia is the most significant associated symptom in patients with this condition [5, 6, 7, 8]. 

The patient in this report had onset of trichotillomania at a very early age (2 years) which has been reported, though rarely. Contrary to the usual cases, where these children have behavioral disturbances and failure to thrive, our patient showed normal physical and mental health. She started pulling and eating her hairs at the age of two years and left after 18months without any specific reason or change of environment, which shows that it is not always the underlying behavioral or psychological disturbance that leads to this habit. This could be “short term habit of hair pulling” which is quite different from trichotillomania. Jealously with the other siblings is a normal phenomenon (as the psychiatrist pointed out in our case) [9]. 

Isolated intestinal trichobezoars are rare but do occur and can have delayed presentation, even after many years of leaving trichophagia. In the index case the trichobezoar might remained in the stomach for years and then dislodged from stomach and stuck in the small bowel, therefore, presented with the acute intestinal obstruction. It is also an interesting fact that in the stomach the trichobezoars do not produce any kind of symptoms for years.

To conclude, delayed presentation of trichobezoar is a rare event. Ileal trichobezoar should be placed in differential of mobile abdominal masses in young girls.

## Footnotes

**Source of Support:** Nil

**Conflict of Interest:** None declared
